# Terminology extraction from medical texts in Polish

**DOI:** 10.1186/2041-1480-5-24

**Published:** 2014-05-31

**Authors:** Małgorzata Marciniak, Agnieszka Mykowiecka

**Affiliations:** 1 Institute of Computer Science PAS, Jana Kazimierza 5, 01-248 Warsaw, Poland

## Abstract

**Background:**

Hospital documents contain free text describing the most important facts relating to patients and their illnesses. These documents are written in specific language containing medical terminology related to hospital treatment. Their automatic processing can help in verifying the consistency of hospital documentation and obtaining statistical data. To perform this task we need information on the phrases we are looking for. At the moment, clinical Polish resources are sparse. The existing terminologies, such as Polish Medical Subject Headings (MeSH), do not provide sufficient coverage for clinical tasks. It would be helpful therefore if it were possible to automatically prepare, on the basis of a data sample, an initial set of terms which, after manual verification, could be used for the purpose of information extraction.

**Results:**

Using a combination of linguistic and statistical methods for processing over 1200 children hospital discharge records, we obtained a list of single and multiword terms used in hospital discharge documents written in Polish. The phrases are ordered according to their presumed importance in domain texts measured by the frequency of use of a phrase and the variety of its contexts. The evaluation showed that the automatically identified phrases cover about 84% of terms in domain texts. At the top of the ranked list, only 4% out of 400 terms were incorrect while out of the final 200, 20% of expressions were either not domain related or syntactically incorrect. We also observed that 70% of the obtained terms are not included in the Polish MeSH.

**Conclusions:**

Automatic terminology extraction can give results which are of a quality high enough to be taken as a starting point for building domain related terminological dictionaries or ontologies. This approach can be useful for preparing terminological resources for very specific subdomains for which no relevant terminologies already exist. The evaluation performed showed that none of the tested ranking procedures were able to filter out all improperly constructed noun phrases from the top of the list. Careful choice of noun phrases is crucial to the usefulness of the created terminological resource in applications such as lexicon construction or acquisition of semantic relations from texts.

## Background

Terminology extraction is the process of identifying domain specific phrases (terms) based on the analysis of domain related texts. It is a crucial component of more advanced tasks like: building ontologies for specific domains, document indexing, construction of dictionaries and glossaries. The subject has been undertaken quite often, particularly in the context of molecular biology terminology. In particular, the Medline abstracts database was frequently used as a data source for protein and gene names, [1,2]. The biomedical domain is changing so rapidly that manually prepared dictionaries are becoming outdated very quickly. In more stable domains, like clinical medicine, a lot of terminology also exists which is used locally and which is not listed in any dictionaries. For many languages, medicine and biomedicine terminology is covered by several sources like those available in UMLS [3], e.g. MeSH or SNOMED, but there are still a lot of domain related expressions occurring within clinical texts which are not included there. Moreover, there are a number of languages (like Polish), whose medical linguistic resources are underdeveloped. In particular, for the Polish language there are no computer dictionaries, except MeSH, with medical vocabulary or terminology, nor is there a SNOMED translation.

This lack of resources and the need for keeping up to date resources describing rapidly changing subdomains has lead to exploring the idea of automatic terminology extraction. Several different approaches to this task are discussed in [4]. It may be observed in the research reported there that, regardless of the detailed assumptions undertaken in the particular solutions, terminology extraction usually consists of two steps. The first one identifies candidates for the terms, and is usually supported by linguistic knowledge. The second step, based on statistics, involves ranking and filtering candidates according to some measure of their relative domain importance. Although the general scheme of term extraction is quite stable, the specificity of a particular natural language, the domain of interest, the size of data available and the accessibility of language processing tools, can all influence the results. Until now, there has been no single strategy which can precisely select terms from non terms and which has proved to be best for all the domains and languages tested.

Automatic extraction of phrases from texts makes it possible not only to prepare a list of domain related terms, but also to identify the exact ways in which they are expressed in context. These results can be used later on to help create a domain ontology and in specifying the information that may be extracted from documents with rule based methods, see [5]. While writing extraction rules we just have to describe all the identified phrases. Assigning one semantic concept or ontology class to all lexical paraphrases requires the normalisation step on which all variants are grouped together. In [6] the normalisation procedures are described. The authors consider the conflation of orthography and inflectional variants, as well as lexical synonyms, structural variants of phrases, and recognition of acronyms and abbreviations.

What is common to all domain vocabularies is that the vast majority of terms are noun phrases. Although in some approaches verbal phrases are also taken into account [7], terminology vocabularies usually contain nominalised versions of such terms. Extracting candidates for domain terms can be based on simple n-grams, e.g. [8], but in most approaches, linguistic information is used. Usually only small shallow grammars are defined [9], but sometimes more elaborate linguistic processing is performed—in [7] the terminology extraction was carried out on fully syntactically parsed texts.

While extracting domain terminology we are interested in compound terms which describe precise concepts, e.g. *kość ramienia* ‘humerus’, the concept’s attributes, e.g. *powiększone węzły chłonne* ‘enlarged lymph nodes’ or relationships between two concepts, e.g. *złamanie kości przedramienia* ‘humerus fracture’. These phrases are not only expressing certain domain important concepts or events but can also be used later on to build up a domain model in which we can include the knowledge that lymph nodes can be enlarged and that the bone can be broken. Recognition of complex expressions can entail recognition of shorter phrases which are part of these longer ones.

At the initial stage of candidate selection, the longest sequences matching the set of defined rules are identified. If we are to order phrases using weights based on the number of times they appear in text, we should also analyse phrases which occur inside others. For example, an occurrence of *lewa nerka prawidłowa* ‘left kidney normal’ should also be counted as an occurrence of the phrases: *nerka* ‘kidney’, *lewa nerka* ‘left kidney’ and *nerka pra-wid-łowy* ‘kidney normal’. Another decision to be made is whether to count the occurrences of all nested phrases or only those which occurred at least once as a separate phrase. It may happen that a term which is very important does not occur even once in a given data set.

The preselected set of phrases constitute input data for the term selection algorithm which usually assigns each phrase a numerical value approximating the relative likelihood that the phrase will constitute a domain term. One of the most popular ranking methods, designed specially for recognising multiword terms, is the C/NC method proposed in [9]. This method takes into account phrase occurrences both in isolation and nested inside longer ones, and the different contexts of their appearances. In this method every phrase is assigned a C-value, which is computed on the basis of the number of times it occurs within the text, its length, and the number of different contexts it takes (within noun phrases in which it occurs).

The definition of the C-value coefficient is given below (p – is a phrase under consideration, LP is a set of phrases containing p), r(LP) – the number of different phrases in LP, *l*(*p*) = *log*_2_(*length*(*p*)).

(1) $$\text{C-value(p)}=\left\{\begin{array}{cc} l(p)*(\text{freq}(p)-\frac{1}{r(\text{LP})}\underset{\text{lp}\;\in \;\text{LP}}{\sum }\text{freq}(\text{lp})), & \text{if}\;r(\text{LP})>0,\\ l(p)*\text{freq}(p), & \text{if}\;r(\text{LP})=0 \end{array}\right)$$

Long phrases tend to occur more rarely than shorter ones so the multiplication by the logarithm of length moves them towards the leading positions. If a nested phrase occurs in one context only, its C-value is set to 0 as it is assumed to be incomplete. If a nested phrase occurs in a lot of different contexts, the chance that it may constitute a domain term increases.

A popular modification of the method was aimed at extending the ranking procedure for phrases of the length 1 which originally all get a 0 value. For this purpose, the logarithm of the length for one word phrases (used in the original solution) was replaced with a non-zero constant. In [10], where this method was applied to Spanish texts, the authors initially set this constant to 0.1, but finally set it to 1, arguing that otherwise one word terms would be located too low on the ranking list.

Comparisons to other term extraction methods, performed in [11] among others, showed that in the biomedical domain termhood-based methods outperform unithood-based methods where termhood is defined as a “degree that a linguistic unit is related to domain-specific concepts”, and unithood as a “degree of strength or stability of syntagmatic combinations and collocations” [12]. In [4] the C-value method, which is based on frequency measure, was judged to be better suited for term identification than mutual information or the Dice Factor describing the degree of association measures.

The C-value obtained using the equation cited above reflects only the relationships between the terms themselves. The results can be improved on the basis of the contexts in which the terms occur within texts. In [9] it was suggested that appearing in the same context as highly ranked terms should increase the rank of the candidate term. For example, the frequent statement *nieprawidłowy twór* ‘abnormal formation’ is ranked high, while the rare one *nieprawidłowy cień* ‘abnormal shadow’ has much lower a C-value. Both phrases occurred in the same singular context: *stwierdzono* ‘found’. On this basis, the low mark of the second term can be increased. The idea is realised by the NC coefficient which is counted according to the following equation in which *t* is a candidate term, *C*_*t*_ is a set of distinct contexts of *t*, *f*_*t*_(*b*) is the frequency of *b* occurring as a context of *t* and *weight*(*b*) = *t*(*b*)/*n* where *t(b)* is the number of terms the context word *b* occurs with and *n* is the total number of the terms considered.

(2) $$\begin{array}{ll} \text{NC-value(t)} & =0.8*\text{C-value(t)}\\ & \;+\;0.2\;*\;\underset{b\in C_{t}}{\sum }f_{t}(b)*\text{weight}(b) \end{array}$$

In the original solution contexts were just strings of wordforms surrounding the given phrase within the text. The authors of [10] proposed using lemmas of the surrounding words instead of their forms for processing Spanish, which has different forms of adjectives and nouns according to number and grammatical gender.

Applying the C/NC scheme or another ranking procedure we get an ordered list of the potential terms. We expect that phrases which are not domain relevant or linguistically incorrect are located low on this list and we are not interested in the exact value of the C/NC coefficient of a particular term. Finally, a cut-off value according to a coefficient value or a position on the list is chosen at the final processing stage. A set of phrases which are located above this cut-off constitute the final result of the terminology extraction task. The different extraction methods can be compared on the basis of a percentage of the selected phrases judged as not being terms during the evaluation stage.

## Results and discussion

The term extraction procedure was conducted on two sets consisting of discharge reports from two wards of a Polish children hospital: the allergies and endocrine ward (further referred to as *o1*) and the surgical ward. They consisted respectively of about 78,000 tokens, and over 360,000 tokens. The analysed texts were very concise as physicians reported only the most important facts there. Thus, it occurred that the great majority of the extracted nominal phrases were domain related. But not all of them were equally useful for the given domain, and a shallow grammar also resulted in extracting some sequences which were not correct phrases at all. Thus, the ordering of the results was still an important task. The C/NC method proved able impose an ordering which located important phrases at the beginning of the ranked list, while incorrect phrases were moved towards its end.

The defined grammar together with the procedure of identifying nested phrases identified more than 4100 different nominal phrases (nested or independent) in the *o1* set, more than 7100 in the surgery set and more than 14150 in the both sets combined together. This means that about 1350 of them occurred in both sets (about one third of the smaller set). The number of phrases extracted using the shallow grammar and the distribution of their length and frequencies are given in Tables 1 and 2. About 20% of these phrases are singular words; the largest group of phrases has two elements (38%) while only about 5% have 5 or more words. The average phrase length is equal to 2.5. More than half of the phrases occurred exactly once, while less than 10% of them occurred more than 10 times.

**Table 1:** Distribution of phrase lengths

**Phrase**	**Data**			**Common**	
**length**	***o1***	**surgery**	***o1*** **+surgery**	**nb**	**% from *o1 * in surg.**
∑	4156	11354	14156	1354	32.58
1	1381	2219	2880	720	52.14
2	1644	4212	5403	453	27.55
3	801	2941	3605	137	17.10
4	242	1301	1511	32	13.22
5	68	476	534	10	14.71
> 5	20	205	223	2	10.00
Max	12(8)	5(7)	12(8)	0	-

**Table 2:** Distribution of phrase frequencies

**Phrase**	**Data**		
**freq**	***o1***	**surgery**	***o1*** **+surgery**
∑	4156	11354	14156
=1	2272	7120	8211
2–10	1417	4076	4572
11–50	325	922	969
51–100	71	115	157
101–1000	71	168	217
1000-	0	28	30

Table 3 shows the distribution of the C-value. About one third of phrases got a 0 value because they always had the same context (within a phrase as its nested subphrase). The remaining 70% of phrases contained correct clinical terms located both at the top of the list as well as close to the bottom of the list. Medical terms which occurred very few times in isolation got a very low positive C-value, e.g. *anestezjolog* ‘anaesthetist’, *torbielka* ‘small cyst’. They cannot be differentiated by the method from nouns such as *kwiat* ‘flower’ or *chodnik* ‘pavement’ which also occurred within the data. The positive effect of counting occurrences of nested phrases can be observed for *ostry dyŻur* ‘emergency service’, for example, which occurred in isolation only once, but was used 82 times in 6 different contexts and classified in 148th place.

**Table 3:** Standard C-value distribution

**Terms**	**Data**		
**freq**	***o1***	**surgery**	***o1*** **+surgery**
∑	4156	11354	14156
C = 0	1110	3458	4163
C > 0	3046	7896	9993
0 < *C* < 1	893	1509	1936
C = 1	565	1301	1708
C > 1	1588	5086	6349
1 < *C* <= 2.5	898	2842	3531
C > 2.5	690	2244	2818

The answer to the question whether to count occurrences of nested phrases which never occur in isolation is not clear. One of the examples of the successful recognition of such a term is *kość ramienna* ‘humerus’. Another example is *miedniczka nerki* ‘renal pelvis’ which also did not occur in isolation but had 15 occurrences in 6 different contexts and was located in 705th place. However, the strategy of promoting nested phrases on the basis of the occurrences of the phrases they are part of, can sometimes lead to undesirable results. The phrase *infekcja dróg* ‘tract infection’ never occurred alone but had 11 different contexts and was located very high (216) in spite of being an incorrect (truncated) phrase. An extreme example of such a phrase which gained a very high C-value is *karta informacyjna leczenia* ‘treatment information card’ being a subsequence of the phrase *karta informacyjna leczenia szptialnego* ‘hospital treatment information card’. In surgical data it occurred 1164 times in this phrase and once in a longer phrase *poprzednia karta informacyjna leczenia szpitalnego* ‘previous hospital treatment information card’. For the C-value counting algorithm this meant there were two different contexts in which this phrase appeared, and resulted in the sixth top value for a phrase which did not occur in the data and is probably not used at all.

The equation for C-value promotes sequences which have different contexts but, in the case of nested phrases, it may be possible that all these contexts describe a super phrase. e.g. for *klatka*_*s* *u* *b* *s* *t*_ (‘cage’, ‘case’, ‘frame’) there are several context super phrases like: *klatka*_*s* *u* *b* *s* *t*_*piersiowa*_*a* *d* *j*_ ‘chest’, *USG klatki piersiowej* ‘chest ultrasound’, *RTG klatki piersiowej* ‘chest RTG’, *zdjęcie klatki piersiowej* ’chest picture’, *klatka piersiowa prawidłowa* ‘chest normal’, but all these are contexts for the term *klatka piersiowa* ‘chest’ and should not promote *klatka* as an independent term. This word is ambiguous and is rather rarely used alone with respect to *klatka piersiowa* ‘chest’. The accepted solution (named as C _1_) relies on counting super phrases which differ only in the words adjacent to a given term.

The distribution of the C _1_-value is given in Table 4. For the C _1_-value method the phrase: *karta informacyjna leczenia* ‘treatment information card’, which occurred only as the nested phrase and has only one context, obtained the proper 0 C _1_-value. The proposed strategy, however, did not eliminate all “unfinished” phrases and yielded only a slight lowering of their score, e.g. from 28th place down to 45th for *USG jamy* ‘USG of cavity’ in the list for surgical data. The high ranking of this phrase on the terminology list is a result of it being part of the following two phrases: *USG*_*b* *r* *e* *v*:*n* *w*_*jamy *_*s* *u* *b* *s* *t*:*g* *e* *n*_*brzusznej*_*a* *d* *j*:*g* *e* *n*_ (used 377 times alone and 51 as a nested phrase) and less common *USG*_*b* *r* *e* *v*:*n* *w*_*jamy*_*s* *u* *b* *s* *t*:*g* *e* *n*_*brzucha*_*s* *u* *b* *s* *t*:*g* *e* *n*_ (used 3 times alone). Both phrases have the same English equivalent: ‘USG of abdominal cavity’. Moreover, the phrase *USG jamy* was recognised once in isolation because of a spelling error in the word *brzusznej* ‘abdominal’.

**Table 4:** **C**_**1**_**-value distribution**

**Terms**	**Data**		
**freq**	***o1***	**surgery**	***o1*** **+surgery**
∑	4156	11354	14156
C = 0	2843	4140	4933
C > 0	2843	7214	9223
0 < *C* < 1	775	1243	1625
C = 1	581	1339	1757
1 < *C* <= 2.5	843	1487	3227
C > 2.5	644	2068	2614

C _1_ coefficients are by definition usually lower than the original C-values. However, the changes in the ranking order are not very large. For *o1* data, of the top 600 elements 20 received a C _1_-value equal to 0. Only two of them were good medical terms, the rest were incomplete phrases like the one described above and were correctly suppressed. For surgical data, these extreme changes were even smaller—4 in 600 top phrases got a 0 C _1_-values, one of them is a correct medical term. In the entire surgical data, 119 terms which had a non-zero C-value got a 0 C _1_-value, 46 of them were incorrect phrases. For the previously given example, *infekcja dróg*, we got 4 contexts instead of 11, the coefficient value was lowered by about 20%, but the position changed only by 20. Similarly, for the very frequent phrase *USG jamy* the change, equal to about 40% of coefficient value, resulted in a small change in position (of 17 places).

In order to identify terminology that may not be related to the medical domain, we compared the terminology extracted from medical data with phrases extracted from the general corpus of the Polish language (National Corpus of Polish (NKJP) [13])—processed and ranked using the same tools. Then we compared terminology identified in NKJP and medical data: surgery and *o1* separately. Table 5 shows how many terms are recognised in both corpora (NKJP and the medical one) and the number of terms that have a higher C _1_-value in the NKJP data. This comparison gives only a general overview as the sizes of the compared corpora are different. The longest common phrase has four words and there is only one in both corpora *infekcja górnych dróg oddechowych* ‘upper respiratory tract infection’. Multi-word terms that have a C _1_-value higher in the NKJP data account for about 2% of multi-word terms for *o1* data and less than 1% for surgery data. Moreover, most multi-word terms with a higher C _1_-value in NKJP are related to the medical domain, e.g.: *poradnia zdrowia psychicznego* ‘mental health clinic’, *przewód pokarmowy* ‘gastrointestinal tract’, *oddział intensywnej terapii* ‘intensive care unit’. But, of course, there are also terms that are common in everyday language like: *numer telefonu* ‘telephone number’, *drugie danie* ‘second course’ or *wycieczka autokarowa* ‘bus trip’. The comparison shows that in hospital documents there are very few phrases that are frequently used in the corpus of general Polish. Moreover, the common phrases are usually related to medicine. So, this stage turned out not to substantially influence the results.

**Table 5:** Comparison with general corpus

**Terms**	***o1***	**Surgery**
Common with NKJP	791	1155
1-word	680	969
Multi words	111	186
C _1_-value greater in NKJP	431	546
1-word	374	477
Multi words	57	69

Finally we ordered the terms according to the C _1_/NC method. Tables 6 and 7 shows the leading terms for both data sets.

**Table 6:** **Top 20 phrases in *o1 * data**

**Phrase**	**C**_**1**_**/NC**	**Full**	**Nested**
*karta informacyjna leczenia szpitalnego* ‘hospital treatment	185.60	116	0
information card’			
*morfologia krwi* ‘full blood count’	124.00	155	4
*wynik badania* ‘examination result’	114.04	118	27
*masa ciała* ‘body mass’	107.82	122	17
*stan ogólny* ‘general condition’	102.66	75	62
*układ kielichowo-miedniczkowy poszerzony* ‘widened pyelocalyceal system’	102.17	55	0
*pediatria ogólna* ‘general paediatrics’	93.60	117	0
*oddział alergologii* ‘allergy ward’	93.60	117	0
*kod pacjenta* ‘patient code’	92.80	116	0
*USG jamy brzusznej* ‘ultrasound of the abdominal cavity’	92.14	66	10
*lekarz prowadzący* ‘attending physician’	91.28	114	0
*ordynator oddziału* ‘head of hospital department’	91.28	114	0
*badanie ogólne* ‘general examination’	79.51	93	9
*RTG klatki piersiowej* ‘chest X-ray’	78.14	52	12
*nerka prawidłowej wielkości* ‘kidney of normal size’	74.81	59	0
*pęcherzyk Żółciowy prawidłowy* ‘normal gall bladder’	73.54	58	0
*układ kielichowo-miedniczkowy* ‘pyelocalyceal system’	69.35	4	59
*pęcherz moczowy wypełniony* ‘filled bladder’	62.56	42	11
*klatka piersiowa* ‘chest’	58.80	1	87
*badanie* ‘examination’	55.20	35	665

**Table 7:** Top 20 phrases in surgical data

**Phrase**	**C**_**1**_**/NC**	**Full**	**Nested**
*karta informacyjna leczenia szpitalnego* ‘hospital treatment	1862.40	1164	1
information card’			
*oddział chirurgiczno-urazowy* ‘surgical and casualty ward’	1332.80	833	0
*badanie ogólne* ‘general examination’	1030.95	1170	112
*wynik badania* ‘examination result’	964.56	1167	43
*oddział chirurgii* ‘surgical ward’	943.26	1179	3
*kod pacjent* ‘patient code’	931.20	1164	0
*zalecenie lekarskie* ‘medical recommendation’	924.80	1156	0
*zastosowane leczenie* ‘applied treatment’	735.22	919	1
*odpływ pęcherzowo-moczowodowy* ‘vesicoureteral reflux’	678.09	124	317
*pęcherz moczowy* ‘bladder’	662.48	325	525
*wskaźnik protrombinowy* ‘prothrombin ratio’	609.60	762	1
*stan ogólny dobry* ‘good general condition’	526.40	414	0
*grupa krwi* ‘blood group’	520.80	649	4
*USG jamy brzusznej* ‘ultrasound of the abdominal cavity’	511.34	377	51
*układ kielichowo-miedniczkowy* ‘pyelocalyceal system’	508.30	67	267
*karta informacyjna* ‘information card’	470.00	1	1173
*wsteczny odpływ pęcherzowo-moczowodowy* ‘vesicoureteral reflux’	468.70	238	14
*leczenie szpitalne* ‘hospital treatment’	466.40	0	1166
*stan ogólny* ‘general condition’	430.81	222	422
*nerka prawidłowej wielkości* ‘kidney of normal size’	410.84	324	1

To check if the changes introduced by the NC correction method were significant we used the top 300 as a set of terms whose contexts were taken into consideration while calculating the NC coefficient. Unfortunately, clinical notes mostly contain noun phrases and a lot of terms just have punctuation marks as their contexts. Thus, reordering phrases according to the NC values did not introduce many changes. In fact, most corrections only caused a difference of no more than 20 places. The bigger differences were seen only at the bottom of the list where they are not very important, as usually, the end of the list is not taken into account as a source of domain terms. The possible explanation of this minor positive effect is the relatively small size of the available data, as some phrases from this 300 element list occurred little more than 15 times.

### Manual evaluation

We performed two tests to evaluate the results of the extraction procedure. The first test was aimed at checking the completeness of the initial list of all considered nominal phrases. It involved the manual identification of terminology in documents and checking how many of these terms were present in the full list of terms before truncating it. The *o1* documents were approximately two times longer, so we randomly selected two (1667 tokens) and four (2074 tokens) documents for the evaluation respectively. The test was performed by two annotators. The results are given in Tables 8 and 9. As is evident from the information in the tables, about 85% of phrases indicated by the annotators are common for both of them. The lists of extracted terms contain above 80% of phrases indicated by the annotators.

**Table 8:** **Phrases in *o1 * texts**

	**1st annot.**	**2nd annot.**	**Common**
nb of phrases	241	235	208
nb of extr. phr.	199	190	175
% of extr. phr.	82.5	80.0	84.1

**Table 9:** Phrases in surgery texts

	**1st annot.**	**2nd annot.**	**Common**
nb of phrases	163	164	138
nb of extr. phr.	134	136	116
% of extr. phr.	82.2	82.9	84.0

The second test indicated how many medical phrases were at the top, in the middle and at the bottom of the lists of terms ordered from the highest to the lowest score of their C _1_/NC-value. The phrases were judged by the same two annotators, as to whether they belong to the terminology or not. The results of the evaluation are given in Tables 10 and 11. In the top part of the lists, the great majority of terms (about 88%) is judged to be domain related by both annotators. The percentage of badly structured terms is below 10%. The proportion of badly structured terms in the other two sets is evidently higher which proves that the C/NC ranking method moves bad terms toward the end of the list. However, as can be seen, even the last section of the list contains 60–82% of domain terms.

**Table 10:** **Phrases considered as terms in *o1 * documents**

	**C**_**1**_**/NC -*o1***											
	**1st annot.**						**2nd annot.**					
	**Domain**		**General**		**Bad**		**Domain**		**General**		**Bad**	
	**nb**	**%**	**nb**	**%**	**nb**	**%**	**nb**	**%**	**nb**	**%**	**nb**	**%**
top200	176	88	19	9.5	5	2.5	178	89	14	7	8	4
middle100	88	88	5	5.0	7	7.0	83	83	8	8	9	9
end100	75	75	18	18.0	7	7.0	82	82	10	10	8	8

**Table 11:** Phrases considered as terms in surgery documents

	**C**_**1**_**/NC - surgery**											
	**1st annot.**						**2nd annot.**					
	**Domain**		**General**		**Bad**		**Domain**		**General**		**Bad**	
	**nb**	**%**	**nb**	**%**	**nb**	**%**	**nb**	**%**	**nb**	**%**	**nb**	**%**
top400	353	88.3	28	7.0	19	4.7	348	87.0	27	6.7	25	6.3
middle200	136	68.0	11	5.5	43	21.5	145	72.5	14	7.0	41	20.5
end200	127	63.5	33	16.5	40	20.0	121	60.5	35	17.5	44	22.0

### Comparison with MeSH

MeSH is a controlled biomedical vocabulary that was created to index articles from biomedical journals and to make literature searches easier. Thus, for example, the data contains the following terms: ‘kidney’ and ‘gallbladder’ but does not contain the phrases: ‘left kidney’ or ‘normal gallbladder’ which are used in hospital documentation but do not function as keywords in journal papers. Experiments in applying MeSH to clinical data were done for English [14] and Swedish [15], UMLS resources were used for information extraction in French [16,17], German [18], and Dutch [19]. A better source of data that contains clinical terminology is SNOMED but it is not translated into Polish. As there are no other publicly available electronic resources of Polish medical terminology we compared the results obtained in the task with the terminology represented in the Polish MeSH thesaurus. We performed the experiment on the version available from http://www.nlm.nih.gov/mesh/ updated in 2012 which contains 26581 main headings and 17638 synonyms. The data is being created in the GBL (Central Medical Library) in Warsaw.

The extracted terms have simplified base forms which cannot be directly compared with the thesaurus that contains terms in their nominative base form. There are three possible solutions to this problem. The first one is to convert the terminology from simplified base forms into correct grammatical phrases and check them in MeSH. The second approach consists in converting MeSH data into simplified base forms. The third approach is to compare the simplified forms with data in MeSH using approximate string matching.

We tested the first and the last method described above to perform a comparison of the top ranked surgical ward terminology with the MeSH thesaurus. We wanted to test only medical terminology so we selected 353 terms that underwent positive manual verification by the first annotator. 52 terms (15%) are present in the MeSH thesaurus in their exact form, while 90 (25.5%) exact forms are nested in other terms. The method for approximate string matching performed on the simplified forms increased the number of recognised terms to 106 (30%). 9 terms recognised by the method using exact forms were not recognised by the last method. Almost all these phrases contain gerunds whose lemma forms differ significantly from the words, e.g: *leczenie*_*g* *e* *r*_*szpitalne*_*a* *d* *j*_ ‘hospital treatment’ has a simplified base form *leczyć szpitalny*. Finally, we tested the approximate string matching method on the set of terms consisting of grammatical phrases. In this case 119 (34%) terms gave positive results.

The results presented in this paper are worse than the results discussed in the paper [20]. In that experiment from 1987, manually extracted terminology from hospital documents was compared with the English MeSH. The authors concluded that about 40% of these phrases were present in MeSH. The results we obtained are even worse and they show that the Polish MeSH is not large enough for the evaluation of clinical terminology extracted from hospital documentation, so in this task it cannot serve as a source of normalised terminology.

### Results for simplified grammar

Finally, we tested whether the precision of the extraction grammar influences the results. We performed an experiment in which we changed the grammar used for phrase identification in such a way that it relied only on information about part of speech and did not take into account gender, number and case agreement. Polish taggers are not very reliable in assessing detailed values of morphological tags, especially for domain specific text, while preparation of correction rules is time consuming. However, neglecting this information results in the extraction of many phrases that are syntactically incorrect. The experiment performed on the surgical data resulted in obtaining 13591 candidates (compared to 11354). Although the results (see Table 12) obtained for the first 400 terms were good – 87.5% of terms were classified as domain related (in comparison to 88.3% obtained with the original grammar), but in the next 400 places the changes were more significant: only 77.5% of the terms were domain related while 18.75% were badly structured (82.8% and 12.5% for the original grammar). These results confirm the hypothesis that better initial selection of candidates has a positive impact on the final results of the chosen method of terminology ranking.

**Table 12:** Comparison of the results for different grammars for surgery documents

	**C**_**1**_** - surgery**											
	**Original grammar**						**Simplified grammar**					
	**Domain**		**General**		**Bad**		**Domain**		**General**		**Bad**	
	**nb**	**%**	**nb**	**%**	**nb**	**%**	**nb**	**%**	**nb**	**%**	**nb**	**%**
top400	353	88.3	28	7.0	19	4.7	350	87.5	19	4.75	31	7.75
next400	331	82.8	19	12.5	50	12.5	310	77.5	15	3.75	75	18.75

## Conclusions

The analysis of the results obtained in the automatic terminology extraction showed that the top part of the terminology list contains phrases that refer almost unexceptionally to the most frequent domain related concepts described in the data. The extracted terms may help to create a domain ontology and, most importantly, they reflect the variety of phrases that are used in everyday hospital practice. The method can be useful for preparing terminological resources for very specific subdomains for which no relevant databases already exist.

Clinical texts contain practically only domain specific knowledge and almost all correct phrases extracted by the grammar are domain related. Thus, the standard method of filtering the results by comparing the occurrences of phrases to their frequencies in the general corpora cannot improve the results. As multiword expressions are less likely to be ambiguous for some domains, general data can be used as an additional source of information about possible contexts.

The C-value approach turned out to be useful for recognizing terms being subsequences of other phrases. The performed evaluation showed that none of the tested ranking procedures were able to filter out all improperly constructed noun phrases from the top of the list, so the processing stage consisting in choosing noun phrases turned out to be very important to the usefulness of the created terminological resource.

In particular, the comparison of the obtained results with manually extracted terminology from selected documents showed that proper morphological tagging is very important to the selected approach. The application of the NC part of the C/NC method to the clinical data does not significantly change the order of terms, so the NC step is not very useful if the aim is to collect all possible domain related phrases, but can help in selecting those that are most important in a particular domain.

## Methods

### Text characteristics

We analysed two sets of data containing hospital discharge documents. They were collected from two wards of a children’s hospital. The first set of data consisted of 116 documents (about 78,000 tokens) relating to patients with allergies and endocrine diseases. The second data set contained 1165 documents from a surgical ward (more than 360,000 tokens). The documents were originally written in MS Word. They were converted into plain text files to facilitate their linguistic analysis. During conversion, information serving identification purposes was substituted with symbolic codes. The vocabulary of the clinical documents is very specific, and significantly differs from general Polish texts. In medical data there are many abbreviations and acronyms, some of them are in common use: *RTG* ‘X-ray’ or *godz* (*godzina*) ‘hour’, but many of them are domain dependent. For example, *por.* in everyday language means *porównaj* ‘compare’, but in the medical domain it is more often the abbreviation for *poradnia* ‘clinic’. Some abbreviations are created ad hoc, e.g., in the phrase *babka lancetowata* ‘ribwort plantain’ the word *lancetowata* ‘ribwort’ is abbreviated to *lan* or *lanc*. These abbreviations cannot be properly recognised out of context. Moreover, many diagnoses or treatments are written in Latin, e.g., *immobilisatio gypsea* ‘immobilisation with gypsum’.

Another problem in analysing clinical data is misspelled words. As the notes are not meant to be published, the texts are not very well edited. Despite the spelling correction tool being turned on, some errors still occurred, mainly in words missed from the standard editor dictionary like *echogeniczności* ‘echogenicity’ misspelled as *echiogeniczności, echogenicznosci* and *echogenicznośąci*. Grammatical errors are infrequent but most utterances are just noun phrases, not complete sentences. Thus, our observations concerning the overall linguistic characteristics of Polish clinical data are consistent with those described by Kokkinakis and Thurin for Swedish [15].

The first level of the linguistic analysis of data is its segmentation into tokens. At this level we distinguish: words, numbers and special characters. Words and numbers cannot contain any special characters. Words may contain digits, but they do not start with digits. So, the string *12mm* is divided into 2 tokens: *12*—number and *mm*—word, while the string *B12* is treated as one word.

In the next step of data processing we annotated the data with morphological information. Each word was assigned its base form, part of speech, and complete morphological characteristics. The annotation is done by the TaKIPI tagger [21] that cooperates with the Morfeusz SIAT morphological analyser [22] and the *Guesser* module [23] that suggests tags for words that are not in the dictionary.

To correct *Guesser*’s suggestions and some systematic tagging errors, we manually prepared a set of global correction rules that work without context, see [24], so they were only able to eliminate some errors, e.g. replace very unlikely interpretations of homonyms. We also prepared a list of the most common abbreviations, which were assigned the appropriate full form as their lemma. Finally, we (automatically) removed improperly recognised sentence endings after abbreviations, and added the end of sentence tags at the ends of paragraphs.

### Phrase selection

In this work we decided only to analyse nominal phrases and put verbal constructions aside. The internal syntactic structure of nominal phrases that constitute terms can vary, but not all types of nominal phrases’ structures are likely to characterise terminological items. In Polish, domain terms most frequently have one of the following syntactic structures: 

• a single noun or an acronym, e.g. *angiografia* ‘angiography’, *RTG* ‘X-ray’;

• a noun followed (or, more rarely, preceded) by an adjective, e.g. *granulocyty*_*s* *u* *b* *s* *t*_*obojętnochłonne*_*a* *d* *j*_ ‘neutrofils’, *ostry*_*a* *d* *j*_*dyŻur*_*s* *u* *b* *s* *t*_ ‘emergency service’;

• a sequence of a noun and another noun in genitive, e.g. *biopsja*_*s* *u* *b* *s* *t*:*n* *o* *m*_*tarczycy*_*s* *u* *b* *s* *t*:*g* *e* *n*_ ‘biopsy of thyroid’;

• a combination of the last two structures, e.g. *gazometria*_*s* *u* *b* *s* *t*:*n* *o* *m*_*krwi*_*s* *u* *b* *s* *t*:*g* *e* *n*_ tętniczej _*a* *d* *j*:*g* *e* *n*_ ‘arterial blood gasometry’.

The syntactic rules become more complicated as one wants to take additional features of Polish nominal phrases into account: 

• word order: as Polish is a relatively free order language, order of phrase elements can vary;

• genitive phrase nesting: the sequences of genitive modifiers can have more than two elements, e.g. *wodonercze*_*s* *u* *b* *s* *t*:*n* *o* *m*_*niewielkiego*_*a* *d* *j*:*g* *e* *n*_*stopnia*_*s* *u* *b* *s* *t*:*g* *e* *n*_*dolnego*_*a* *d* *j*:*g* *e* *n*_*układu*_*s* *u* *b* *s* *t*:*g* *e* *n*_*podwójnego*_*a* *d* *j*:*g* *e* *n*_*nerki*_*s* *u* *b* *s* *t*:*g* *e* *n*_*prawej*_*a* *d* *j*:*g* *e* *n*_ ‘mild hydronephrosis of the duplicated lower collecting system of the right kidney’;

• coordination: some terms include coordination (of noun or adjectival phrases), eg. *USG naczyń szyjnych i kręgowych* ‘ultrasound of the carotid and vertebral vessels’, *zapalenie mózgu i rdzenia* ‘inflammation of brain and medulla’;

• prepositional phrases: there are also terms like *witaminy z grupy B* ‘vitamins of the B group’ which include prepositional phrases inside.

In our work we account for all of the nominal phrase types described above, except those including prepositional phrases and nominal coordination. To recognise them, we defined a shallow grammar consisting of a cascade of six sets of rules being regular expressions. The rules operate on the data annotated with a part of speech and the values of morphological features. The results obtained by applying a set of rules on one level were used as the input for the subsequent set. The rules are cited in Table 13 in a format slightly modified for this presentation; in particular, this format does not include the output part of the rules. Indexes describe values of morphological features. Names in lowercase correspond to the respective feature values, capitalized names correspond to variables referring to case (C, C2), gender (G, G2) or number (N, N2).

**Table 13:** The sets of rules for recognizing noun phrases

**Set**	**Rules**
I	N subst | ger
	NC (foreign_subst | foreign) +foreign?+foreign?
	NC brev _*n* *p* *u* *n*,*n* *w*_| brev _*n* *p* *u* *n*,*n* *w*_
	NC brev _*p* *u* *n*,*n* *w*_ + “.”? | brev _*p* *u* *n*,*n* *w*_ + “.”?
	NC brev _*n* *p* *u* *n*,*n* *p* *h* *r*_| brev _*n* *p* *u* *n*,*n* *p* *h* *r*_
	NC brev _*p* *u* *n*,*n* *p* *h* *r*_ + “.”? | brev _*p* *u* *n*,*n* *p* *h* *r*_ + “.”?
	AJ ^2 adv?+(adj _*C*,*G*,*N*_| ppas _*C*,*G*,*N*_)
	AC brev _*a* *d* *j* *w*,*n* *p* *u* *n*_| brev _*a* *d* *j* *w*,*p* *u* *n*_ + “.”?
	CN “i”
II	A AJ+adv?
	A ^3 AC + “-” + AJ _*C*,*G*,*N*_
	A ^3 adja + “-” + AJ _*C*,*G*,*N*_
	AC AC + “-”+AC
	N N _*C*,*G*,*N*_+AJ _*C*,*G*,*N*_| AJ _*C*,*G*,*N*_+N _*C*,*G*,*N*_
	NZ subst(lemma=to/co/obrąb/kierunek/cel/czas/ moŻliwość/podstawa/ciąg/cecha/...)
	AZ IR(lemma=aktualny/daleki/gdy/pewien/wzgląd/ ten/inny/sam/niektóry/wczesny/...)
III	ADJP A
	ADJP A _*C*,*G*,*N*_?+A _*C*,*G*,*N*_?+A _*C*,*G*,*N*_?+A _*C*,*G*,*N*_?+CN+A _*C*,*G*,*N*_
	ADJP A _*C*,*G*,*N*_?+A _*C*,*G*,*N*_?+A _*C*,*G*,*N*_?+A _*C*,*G*,*N*_+AC?
IV	NB ^2 NC+ADJP
	NB ^2 AC+N
	NB N+AC
	NB ADJP _*C*,*G*,*N*_+NC
	NB ADJP _*C*,*G*,*N*_?+N _*C*,*G*,*N*_+ADJP _*C*,*G*,*N*_?
V	NG NB _*g* *e* *n*_?+NB _*g* *e* *n*_?+NB _*g* *e* *n*_?+NB _*g* *e* *n*_?+CN?+NB _*g* *e* *n*_
	NG NB
VI	X NG+NG _*g* *e* *n*_?+ADJP _*C*,*G*,*N*_?
	X NG+NC

The Polish tagset is quite detailed (over 1000 actually used tags) and contains around 30 word classes. This set, for our purposes, was extended by the *foreign* tag used for Latin or English words used in discharge summaries. Words which can build up a nominal phrase can be from one of the following categories: *subst (noun), ger (gerund), foreign_subst, foreign*, and *brev:pun:nw, brev:pun:nphr, brev:npun:nw, brev:npun:nphr* (abbreviation/acronym of a noun or noun phrase requiring or not requiring a period afterwards). The first two types of these core elements inflect and they are assigned to the N class. Foreign words and abbreviations do not inflect but they can also be modified by adjectives. These words cannot be a source of gender, number or case values and are assigned the category NC. Foreign names frequently consist of more than one element, so sequences of up to three foreign words are also accepted by the grammar (we do not analyse the internal structure of Latin or English sequences). The first set of rules also includes rules for identifying basic adjectives—inflective (AJ) and non-inflective (AC) which can possibly be modified by adverbs. The ^*X* notation is used to mark cases in which the morphological description of the resulting phrase should be copied from the Xth element of the rule and not from the first one (e.g. case, gender and number of an adjective phrase consisting of an adverb and an adjective should be the same as those of the adjective).

In the second set of rules, adverbs can be attached to adjectives which are in front of them (but only if there is no adjective after them—this more preferable attachment is covered by the first set of rules). There are also rules for special types of Polish complex adjectives—constructions like *pęcherzowo-moczowodowy* ‘vesico-ureteric’ containing a special form of an adjective ending with “-o” followed by a hyphen and an adjective. The last two rules of the second set are defined specially for the procedure of nested phrases’ borders identification procedure (special rules are responsible for not constructing nested phrases which include adjectives but do not include the nouns they modify).

The third set of rules describes compound adjectival phrases, the fourth one combines adjectival phrases with nouns, the fifth describes sequences of genitive modifiers, and the last one combines genitive modifiers and optional adjectival modifiers which can occur after genitive ones. There is also a rule which allows for a non-inflective noun as a last phrase element. This rule accounts for acronyms used at the end of noun phrases, but it turned out that due to the lack of punctuation it was responsible for recognising improperly structured phrases.

Applying such a general set of rules to our data would result in a subset of phrases which we considered non-domain terms. These were phrases beginning with modifiers describing that a concept represented by a subsequent nested phrase was occurring, desired or expected, e.g. *(w) trakcie*_*s* *u* *b* *s* *t*_*choroby* ‘during illness’. To eliminate such phrases we defined a set of words which were to be ignored during phrase construction. Rules for recognising them (and assigning NZ or AZ category) were added to the first set. These words belong to the following three classes: 

• general time or duration specification, e.g. *czas* ‘time’, *miesiąc* ‘month’;

• names of months, weekdays;

• introductory/intension specific words, e.g. *kierunek* ’direction’, *cel* ‘goal’, *podstawa* ’base’, *cecha* ‘feature’ (22 words more).

In the results presented in this paper, only some types of normalisation of the extracted terms described in [6] are completed. We recognise morphological variants of terms. Domain abbreviations and acronyms that have a unique interpretation were extended and thus matched with their full versions. This cannot always be done in a straightforward manner, as there are many abbreviations/acronyms that can be correctly interpreted only in context. Moreover, discharge documents do not contain definitions of abbreviations or acronyms, and many acronyms are created from English phrases (e.g. MCV—**M**ean **C**orpuscular **V**olume) so it is impossible to adapt the method proposed in [25] for acronym recognition, which was based on analysing acronym definitions.

### Identification of nested phrases and term weighting

In order to apply the C-value method, the operation of identifying phrases nested within other phrases is crucial. In our solution, borders of nested phrases are introduced by the grammar. As a nested phrase we take every fragment of a nominal phrase which is recognised by any of the grammar rules as being a noun phrase itself. For example, *pęcherzyk*_*s* *u* *b* *s* *t*_*Żółciowy*_*a* *d* *j*_ ‘gall bladder’ usually occurs with an adjective describing its condition e.g, *pęcherzyk*_*s* *u* *b* *s* *t*_*Żółciowy*_*a* *d* *j*_*prawidłowy*_*a* *d* *j*_ ‘normal gall bladder’, or *kość*_*s* *u* *b* *s* *t*_ ramienna _*a* *d* *j*_ ‘humerus’ occurs with information indicating the left or right side. Recognising the first exemplary phrase results in identifying two candidates: *pęcherzyk*_*s* *u* *b* *s* *t*_*Żółciowy*_*a* *d* *j*_*prawidłowy*_*a* *d* *j*_ and *pęcherzyk*_*s* *u* *b* *s* *t*_*Żółciowy*_*a* *d* *j*_ but not *Żółciowy*_*a* *d* *j*_*prawidłowy*_*a* *d* *j*_ as this is not a noun phrase.

The original work in which the C/NC method was proposed concerned English—a language with little inflection and a rather stable noun phrase structure. Thus, the authors did not have to pay a lot of attention to defining how they compared phrases and counted the number of different contexts. They compared word forms. However, for highly inflectional languages, like Polish, different forms of a word can vary significantly, making a decision on term equality harder. Because of this, finding repeated nested phrases also cannot be done by just matching the strings. For example, the following nominal phrase in the nominative (which is traditionally considered a basic form): *zakaŻenie*_*s* *u* *b* *s* *t*:*g* *e* *n*_*wirusem*_*s* *u* *b* *s* *t*:*d* *a* *t*_*grypy*_*s* *u* *b* *s* *t*:*g* *e* *n*_ ‘influenza virus infection’ is written in the genitive as: *zakaŻenia*_*s* *u* *b* *s* *t*:*g* *e* *n*_*wirusem*_*s* *u* *b* *s* *t*:*d* *a* *t*_*grypy*_*s* *u* *b* *s* *t*:*g* *e* *n*_ ‘influenza virus infection’. In this latter phrase we ought to recognise the term *zakaŻenie wirusem grypy* and three nested phrases: *wirus grypy*, *wirus* and *grypa*. None of them directly matches the considered phrase. The first one matches the basic (nominative) form, but the nominative form of the nested phrases does not match either the genitive or nominative form of the entire phrase. This proves that lemmatisation of the entire phrase does not solve the problem.

To overcome this difficulty we decided to transform the identified phrases into simplified base forms, being sequences of lemmas of phrase elements. In the cited example, such a simplified lemma is: *zakaŻenie wirus grypa* ‘infection virus influenza’. In this sequence all the above nested terms (converted into their simplified base forms) can be found easily.

Our approach is much simpler and more robust than a formally correct one. It allows not only for easier recognition of nested phrases but also helps in cases where establishing a correct basic form can be difficult for shallow rules. For example, the correct lemma for the phrase *okresowego*_*g* *e* *n*_*badania *_*g* *e* *n*_*ogólnego*_*g* *e* *n*_*moczu*_*g* *e* *n*_ should be *okresowe badanie ogólne moczu* ‘periodic general examination of urine (periodic urinalysis)’ but could possibly also (syntactically) be *okresowe badanie ogólnego moczu* ‘periodic examination of general urine’. Introducing artificial base forms we avoid this difficulty. Simplified base forms allow us also to join phrases with various abbreviations of the same word like *babka lan* and *babka lanc* with their full form—*babka lancetowata* ‘ribwort plantain’ (from patch tests). As proper lemmatisation of all phrases is also prone to tagging errors, our approach is much easier and more robust than a formally correct one.

The lemmatisation approach explained above means that sometimes semantically different phrases have the same simplified base forms.

This may happen due to: 

• phrases with genitive modifiers occurring in different numbers e.g. *zapalenie ucha* ‘ear inflammation’ and *zapalenie uszu* ‘ears inflammation’ are both converted into the singular;

• the adjectives in different degrees (small, smaller) having the same base forms, e.g. *miednica mała* ‘small pelvis’ (more frequently written as *mała miednica* where *mała* ‘small’ refers to its size) and *miednica mniejsza* (*mniejsza* ‘smaller’ indicates anatomic part) ‘lower pelvis’;

• negated and positive forms of adjectival participles, e.g. *powiększony*/*niepowiększony* ‘increased’/’not increased’, both have the lemma *powiększyć*_*i* *n* *f*_ ‘increase’.

• gerunds and participles having infinitives as their base forms, so e.g.: phrases *usunięcie*_*g* *e* *r*_*kamienia*_*s* *u* *b* *s* *t*:*g* *e* *n*_ ‘removing stone’ (an operation) and *usunięty*_*p* *p* *a* *s*_*kamień*_*s* *u* *b* *s* *t*:*n* *o* *m*_ ‘removed stone’ (description of the stone) have the same simplified base form *usunąć *_*i* *n* *f*_*kamień*_*s* *u* *b* *s* *t*_.

After normalisation of the recognised phrases consisting in their transformation into simplified forms we have to decide on a way of differentiating contexts. The C-value coefficient greatly depends on the way for counting the number of different contexts in which a nested phrase occurs. In comparison to [9], we introduced slight modifications to the way of computing this number. In the original solution all different sequences consisting of different initial words and different final words were counted. For example, if we consider a set of four terms: 

• *powiększenie* [*węzłówchłonnych*] ‘lymph nodes enlargement’

• *powiększenie* [*węzłówchłonnych*] *krezkowych* ‘mesenteric lymph nodes enlargement’

• *znacznepowiększenie* [*węzłówchłonnych*] ‘significant lymph nodes enlargement’

• *powiększenie* [*węzłówchłonnych*] *szyji* ‘neck lymph nodes enlargement’

the number of context types for *węzłów*_*s* *u* *b* *s* *t*:*p* *l*:*g* *e* *n*_*chłonnych*_*a* *d* *j*:*p* *l*:*g* *e* *n*_ ‘lymph nodes’ would be four. But this method of context counting obscures the fact that the close context of *węzłów chłonnych* does not change that much. To account for this phenomenon, one may count only the one word context of any nested phrase.

While choosing this option one has still many possibilities to combine right and left contexts. We tested three approaches: the first one was to count pairs of left and right full contexts combined together; in the second approach we counted different words in both left and right contexts grouped together. However, the best results were obtained for the third option in which we took the maximum from different left and right words’ contexts counted separately. So, in the above example, the left context is empty as the same word *powiększenie* ‘enlargement’ appears in all phrases. This version is called C _1_. For our example the number of different contexts calculated using these methods would be accordingly: 

4: *powiększenie, powiększenie-krezkowych, znaczne-powiększenie, powiększenie-szyji*;

3: *powiększenie, krezkowych, szyji*;

2: *krezkowych, szyji*.

We counted the C-value for all phrases including those of length 1. However, we set l(p) in the equation (1) to 0.1 not to 1 like [10]. We observed that although one word terms constituted only 19% of the first 1000 terms in the *o1* data, while on the entire list there were 33% of them (14% and 19% respectively for surgical data), many of the one word terms occurred only once (34% and 37% respectively). Setting l(p) for one word phrases to 1 result in 46% of the first 1000 terms to be of length 1.

For the results obtained using the C _1_ coefficient, we applied the full C/NC method to take the external terms context into account. For calculating the NC coefficient we used one word contexts which were adjectives, nouns and verbs which occurred immediately before or immediately after any term which was in the top 300 positions according to its C-value coefficient.

Depending on the goal, requiring the imposition of greater stress on the recall or precision of the results, the smaller or larger top part of the list ordered by the NC value can be taken as a resulting terminology resource.

### Manual evaluation

The manual evaluation was performed by two annotators: one was a paediatrician specialising in allergology and pulmunology, the second was involved in the experiment, had a computer background and had experience in linguistic and medical data processing.

The two annotators were only given very general instructions to mark a phrase which they thought of as being important in clinical data and which did not include prepositions. The basic problem of this task was to decide what kind of phrases constituted terminology. Sometimes only the boundaries of the phrase indicated by the annotators were different, e.g: in the phrase *na całym ciele* ‘on the whole body’ only *ciało* ‘body’ was recognised by the first annotator, while the second annotator included the word *całe* ‘whole’. Moreover, both annotators had a tendency to indicate phrases that contained coordinations of nouns which were not covered by the grammar, e.g: *Wyniki podstawowych badań morfotycznych i biochemicznych krwi i moczu* ‘The results of basic morphotic and biochemical blood and urine examinations’. The first annotator recognised 42 terms in the *o1* data that were absent from the automatically prepared list for the following reasons: lack of grammar rules recognising the coordination of nominal phrases – 6 errors; lack of other grammar rules – 8; tagging errors – 11; problems with rules containing abbreviations and their tagging – 10; phrases containing time expressions and introductory/intension specific words (e.g: ‘week’, ‘goal’, ‘direction’) – 6.

For the second evaluation experiment for the *o1* data we took the top 200 terms, and randomly selected 100 terms from the middle of the list (C _1_/NC-value ∈(1.0, 2.5 〉) and 100 from the bottom part of the list (C _1_/NC-value ∈〈0.0, 1.0 〉). For surgery data we evaluated the 400 topmost terms and 200 terms from the middle and bottom part of the lists. Then, the phrases were judged by the same two annotators, as to whether they belonged to the terminology or not. Not all phrases from the top part of the lists were classified as terms. Despite attempts to eliminate semantically odd phrases like *USG jamy* ‘USG of cavity’ and *infekcja dróg* ‘infection of tract’ (only in the *o1* data) they still appear in the top part of the lists as they are often in the data and ‘cavity’ and ‘tract’ are part of several well established phrases. Another problem was caused by abbreviations attached to correct phrases like *uraz głowy S* ‘head injury S’ where S is a part of the ICD-10 code of the illness ‘S00’ written with a space between ‘S’ and ‘00’. Our grammar does not exclude such contractions as it is possible that an abbreviation is at the end of a phrase, e.g: *kontrolne badanie USG* ‘control ultrasound examination’.

### Comparison of simplified terms with MeSH

Below we describe three possible solutions for comparing our list of simplified base forms of terms with terminology in MeSH that contains correctly structured nominal phrases in the nominative case. We applied the first and the last method of term forms matching as described below.

The first one is to convert the terminology from simplified base forms into correct grammatical phrases and check them in MeSH. We have to take into account that the general Polish morphological dictionary does not recognise about 18.8% of word-tokens in clinical data, see [24]. In general, the automatic generation of correct base forms from simplified ones is error prone, but the construction of medical phrases is more restricted than for literary language so the results are better. We performed this task with the help of phrases extracted from clinical data, in which we identified fragments that are stable like genitive complements. This solution significantly decreases the role of unknown words. For example in the phrase *wirus *_*s* *u* *b* *s* *t*:*s* *g*:*n* *o* *m*_*Epsteina*_*s* *u* *b* *s* *t*:*s* *g*:*g* *e* *n*_-*Baar*_*s* *u* *b* *s* *t*:*s* *g*:*g* *e* *n*_ ‘Epstein-Barr virus’ the part *Epsteina*_*s* *u* *b* *s* *t*:*g* *e* *n*_-*Baar*_*s* *u* *b* *s* *t*:*g* *e* *n*_ has the same form in all inflected forms of the whole phrase. So it is possible to copy this part from the phrase extracted from the data. We have to take into account that some of the terminology in Polish MeSH is nominal phrases in the plural, e.g. the above phrase is in plural form in MeSH: *Wirusy *_*s* *u* *b* *s* *t*:*p* *l*:*n* *o* *m*_*Epsteina*_*s* *u* *b* *s* *t*:*s* *g*:*g* *e* *n*_-*Baar*_*s* *u* *b* *s* *t*:*s* *g*:*g* *e* *n*_ ‘Epstein-Barr viruses’. This problem can be overcome by generating both singular and plural forms. This will account for medical plurale tantum phrases like *drogi*_*s* *u* *b* *s* *t*:*p* *l*:*n* *o* *m*_*moczowe*_*a* *d* *j*:*p* *l*:*n* *o* *m*_ ‘urinary tract’ that now are improperly lemmatised to a phrase in the singular *droga*_*s* *u* *b* *s* *t*:*s* *g*:*n* *o* *m*_*moczowa*_*a* *d* *j*:*s* *g*:*n* *o* *m*_.

We converted the selected 353 terms into their correct base forms. For the following 11 terms, their base forms were corrected manually as they were unknown to the morphological dictionary and should be inflected: *urodynamiczny* ‘urodynamic’, *przypęcherzowy* ‘paravesical’, *detromycynowy* ‘chloramphenicol’ and *podpęcherzowy* ‘bladder outlet’ and compound words *pęcherzowo-moczowy* ‘vesicoureteral’ (4 terms) and *miedniczkowo-moczowodowy* ‘pelvi-ureteric’ (3 terms).

The second approach consists in converting MeSH data into simplified base forms. This method also has disadvantages as 42% of words contained in MeSH are not represented in the general Polish dictionary that we used for the annotation of our data and which was used to annotate the NKJP corpus [13]. Converting MeSH terminology into simplified base forms does not solve all problems either. For example, Polish MeSH does not contain the phrase: *chirurgia*_*s* *u* *b* *s* *t*_*naczyniowa*_*a* *d* *j*_ ‘vascular surgery’ but it contains *zabiegi*_*s* *u* *b* *s* *t*_*chirurgiczne*_*a* *d* *j*_*naczyniowe*_*a* *d* *j*_ ‘vascular surgery operations’. The English equivalent of the last phrase contains the first phrase but this is not true of the Polish version. The simplified form of the first phrase *chirurgianaczyniowy* is not contained in the simplified version of the last phrase *zabiegchirurgicznynaczyniowy* as the strings *chirurgia* and *chirurgiczny* are different.

The third approach is to compare the simplified forms with data in MeSH using approximate string matching. To apply this method we perform a sort of stemming by removing suffixes indicating cases of nouns and adjectives. Then we apply the Levenshtein distance measure which takes into account the position of a non-matching letter in the analysed word. Words are more similar if differences are found nearer to the end of the word than to the beginning. For each word from a phrase in question we find a set of similar words. Then we look for MeSH terms that contain one similar word for each phrase element.

## Abbreviations

adj: Adjective; brev: Abbreviation; ICD: International Classification of Diseases; gen: Genitive; ger: Gerund; MeSH: Medical Subject Headings; NKJP: National Corpus of Polish; nom: Nominative; nphr: Noun phrase; npun: No punctuation; nw: Noun word; pl: Plural; pun: Punctuation; POS: Part of Speech; sg: Singular; SNOMED: Systematized Nomenclature of Medicine; subst: Substantive; UMLS: Unified Medical Language.

## Competing interests

The authors declare that they have no competing interests.

## Authors’ contributions

MM performed data pre-processing, developed the evaluation scheme of the results, and scrutinised the entire experiment. AM defined the shallow grammar, implemented C/NC methods, and took part in the evaluation. The whole paper was written and corrected by both authors. Both authors read and approved the final manuscript.

## References

[B1] Tanabe L Wilbur WJ Tagging gene and protein names in full text articles Proceedings of the Workshop on Natural Language Processing in the Biomedical Domain 2002 Philadelphia: Association for Computational Linguistics 9 13

[B2] Bunescu R Ge R Kate RJ Mooney RJ Wong YW Learning to extract proteins and their interactions from medline abstracts Proceedings of ICML-2003 Workshop on Machine Learning in Bioinformatics 2003 Washington, DC: The International Machine Learning Society 46 53

[B3] Unified Medical Language System [http://www.nlm.nih.gov/research/umls/]

[B4] Pazienza M Pennacchiotti M Zanzotto F Sirmakessis S Terminology extraction: sn analysis of linguistic and statistical approaches Knowledge Mining Series: Studies in Fuzziness and Soft Computing 2005 Berlin, Heidelberg, New York: Springer-Verlag

[B5] Mykowiecka A Marciniak M Kupść A Rule-based information extraction from patients’ clinical data J Biomed Inform 2009 42 923 936 10.1016/j.jbi.2009.07.007 19646551

[B6] Nenadić G Ananiadou S McNaught J Enhancing automatic term recognition through recognition of variation Proceedings of Coling 2004 2004 Geneva: COLING 604 610

[B7] Savova GK Harris M Johnson T Pakhomov SV Chute CG A data-driven approach for extracting “the Most Specific Term” for ontology development AMIA Annu Symp Proc 2003 2003 579 583 14728239 PMC1480306

[B8] Wermter J Hahn U Massive biomedical term discovery Discovery Science, LNCS 3735 2005 Berlin, Heidelberg, New York: Springer-Verlag 281 293

[B9] Frantzi K Ananiadou S Mima H Automatic recognition of multi-word terms: the C-value/NC-value Method Int J Digit Libr 2000 3 115 130 10.1007/s007999900023

[B10] Barrón-Cedeno A Sierra G Drouin P Ananiadou S An improved automatic term recognition method for Spanish Computational Linguistics and Intelligent Text Processing, LNCS 5449 2009 Berlin, Heidelberg, New York: Springer-Verlag 125 136

[B11] Korkontzelos I Klapaftis IP Manandhar S Reviewing and evaluating automatic term recognition techniques Advances in Natural Language Processing, LNAI 5221 Volume 5221 2008 Berlin, Heidelberg, New York: Springer-Verlag 248 259

[B12] Kageura K Umino B Method for automatic term recognition. A review Terminology 1996 3 259 289 10.1075/term.3.2.03kag

[B13] Przepiórkowski A, Bańko M, Górski RL, Lewandowska-Tomaszczyk B Narodowy Korpus Języka Polskiego 2012 Warszawa: Wydawnictwo Naukowe PWN

[B14] Cooper GF Miller RA An experiment comparing lexical and statistical methods for extracting MeSH terms from clinical free text JAMIA 1998 5 62 75 9452986 10.1136/jamia.1998.0050062 PMC61276

[B15] Kokkinakis D Thurin A Applying MeSH *â“‡* to the (Swedish) clinical domain - evaluation and lessons learned Proceedings of the 6th Scandinavian Health Informatics and the 12th Swedish National Term Conference 2008 Kalmar: Högskolan i Kalmar eHälsoinstitutet 37 41

[B16] Pereira S Neveol A Serrot E Joubert M Darmoni SJ Kerdelhué G Using multi-terminology indexing for the assignment of MeSH descriptors to health resources in a French online catalogue AMIA Annu Symp Proc 2008 2008 586 590 18998933 PMC2656077

[B17] Gerbier S Yarovaya O Gicquel Q Millet AL Smaldore V Pagliaroli V Metzger MH Darmoni SJ Evaluation of natural language processing from emergency department computerized medical records for intra-hospital syndromic surveillance BMC Med Inform Decis Mak 2011 11 50 10.1186/1472-6947-11-50 21798029 PMC3158541

[B18] Daumke P Schulz S Hahn U Markó K Cross-language MeSH indexing using morpho-semantic normalization AMIA Annu Symp Proc 2003 2003 425 429 14728208 PMC1480043

[B19] Hoste V Vanopstal K Lefever E Delaere I Classification-based scientific term detection in patient information Terminology 2010 16 1 29 10.1075/term.16.1.01hos

[B20] Masarie FE Miller RA Medical subject headings and medical terminology: an analysis of terminology used in hospital charts Bull Med Libr Assoc 1987 2 75 89 94 3297223 PMC227622

[B21] Piasecki M Polish tagger TaKIPI: rule based construction and optimisation Task Q 2007 11 1–2 151 167

[B22] Woliński M Trojanowski K, Wierzchoń S, Kłopotek M Morfeusz — a practical tool for the morphological analysis of polish Intelligent Information Processing and Web Mining, IIS:IIPWM’06 Proceedings 2006 Berlin, Heidelberg, New York: Springer-Verlag 503 512

[B23] Piasecki M Radziszewski A Polish morphological guesser based on a statistical a tergo index Proceedings of the International Multiconference on Computer Science and Information Technology — 2nd International Symposium Advances in Artificial Intelligence and Applications (AAIA’07) 2007 Wisła: Polish Information Processing Society 247 256

[B24] Marciniak M Mykowiecka A Towards morphologically annotated corpus of hospital discharge reports in polish Proceedings of BioNLP 2011 2011 Portland: The Association for Computational Linguistics 92 100

[B25] Ananiadou S Nenadić G Automatic acronym acquisition and term variation management within domain specific texts Proceedings of the 3rd International Conference on Language, Resources, and Evaluation (LREC-3) 2002 Las Palmas: European Language Resources Association 2155 2162

